# End-user perspectives to inform policy and program decisions: a qualitative and quantitative content analysis of lifestyle treatment recommendations by adolescents with obesity

**DOI:** 10.1186/s12887-019-1749-3

**Published:** 2019-11-08

**Authors:** M. Kebbe, A. Perez, A. Buchholz, T.-L. F. McHugh, S. D. Scott, C. Richard, M. P. Dyson, G. D. C. Ball

**Affiliations:** 1 Department of Pediatrics, Faculty of Medicine and Dentistry, University of Alberta, Edmonton Clinic Health Academy, 11405 – 87 Avenue, Edmonton, Alberta Canada; 2 Centre for Healthy Active Living, Children’s Hospital of Eastern Ontario, Ottawa, Ontario Canada; 3 Faculty of Kinesiology, Sport, and Recreation, University of Alberta, Edmonton, Alberta Canada; 4 Faculty of Nursing, University of Alberta, Edmonton, Alberta Canada; 5 Department of Agricultural, Food, and Nutritional Science, Faculty of Agricultural, Life and Environmental Sciences, University of Alberta, Edmonton, Alberta Canada

**Keywords:** Adolescent, Diet, Exercise, Pediatric obesity, Sedentary lifestyle, Sleep

## Abstract

**Background:**

Lifestyle modifications represent the first line of treatment in obesity management; however, many adolescents with obesity do not meet lifestyle recommendations. Given that adolescents are rarely consulted during health policy development and in the design of lifestyle interventions, their first-hand experiences, preferences, and priorities may not be represented. Accordingly, our purpose was to explore adolescents’ lifestyle treatment recommendations to inform policy and program decisions.

**Methods:**

Conducted from July 2017 to January 2018, this study adhered to a qualitative, crosslanguage, patient-oriented design. We recruited 19 13–17-year-old adolescents (body mass index [BMI] ≥85th percentile) seeking multidisciplinary treatment for obesity in geographically and culturally diverse regions of Canada. Adolescents participated in one-on-one, in-person, semi-structured interviews in English or French. Interviews were audio-recorded, transcribed verbatim, managed using *NVivo 11,* and analyzed using quantitative and qualitative content analysis by two independent researchers.

**Results:**

Adolescents’ recommendations were organized into five categories, each of which denotes health as a collective responsibility: *(i)* establish parental support within limits, *(ii)* improve accessibility and availability of ‘healthy foods’, *(iii)* limit deceptive practices in food marketing, *(iv)* improve accessibility and availability of varied physical activity opportunities, and *(v)* delay school start times. Respect for individual autonomy and decision-making capacity were identified as particularly important, however these were confronted with adolescents’ partial knowledge on nutrition and food literacy.

**Conclusions:**

Adolescents’ recommendations highlighted multi-level, multi-component factors that influenced their ability to lead healthy lifestyles. Uptake of these recommendations by policy-makers and program developers may be of added value for lifestyle treatment targeting adolescents with obesity.

## Introduction

Pediatric obesity is recognized as a public health priority and is often accompanied by physical, physiological, and psychosocial health risks [1, 2]. Efforts to reverse excess weight and to promote an improved cardiometabolic profile among children and adolescents with obesity emphasize lifestyle and behavioral modifications as the first line of treatment [3]. Adolescent health policies, including those centered around lifestyle, are frequently directed at individual responsibility and choice rather than depicting broader contextual factors, likely contributing to health disparities for adolescents in the distribution, accessibility, and quality of health services [4]. Ecological theory recognizes that individual determinants are shaped by, and in turn shape, broad environmental drivers, including the family, home, school, and community. Accordingly, these environments can be optimized for physical and psychosocial health by local, provincial, and federal policies.

Given that noncommunicable diseases such as obesity, including determinants and consequences, strongly impact adult health, a life-course perspective is gaining attention to nurture current and adult health status [5, 6]. For example, a body of literature points to the tracking of nutrition (food groups and energy yield macronutrients) and physical activity (frequency, intensity, and duration) at moderate-to-strong levels from childhood into adolescence [7] and sedentary behaviors, with the strongest tracking shown for television viewing, at low-to-moderate levels from adolescence into adulthood [8]. Yet, many adolescents with obesity do not meet lifestyle recommendations [9], bringing attention to the barriers that may influence their ability to make and maintain healthy lifestyle habits.

Adolescence, therefore, becomes an important period for health promotion, prevention, and intervention for obesity since it is a time of much experimentation in light of behavioral, cognitive, physiological, and psychosocial growth. In a series of studies we conducted on the adoption of healthy lifestyle habits, we targeted the first-hand perspectives and experiences of adolescents with obesity, many of whom identified a number of challenges that prevented them from achieving a healthy lifestyle such as the convenience of unhealthy foods, negative experiences in physical education classes in school, sleep difficulties, and mental health issues [10]. Growing decision-making capacity, independence from families, integration into social norms, and iterative identity formation place adolescents in an ideal position to become involved in health policy by providing recommendations to navigate identified barriers.

When end-users were involved, policy-making processes showed increased legitimacy, justifiability, and feasibility over policies designed through more traditional, top-down methods [11]. Adolescents may find themselves disadvantaged by lower levels of investment and competition for limited resources in a complex policy environment that draws on adult perspectives in implementing regulations. In turn, this minimizes their concerns, needs, preferences, and priorities and may explain why they are a challenging group to engage in obesity prevention and management initiatives (e.g.*,* low recruitment and initiation, high attrition) [12]. Indeed, previous research has focused on adult consultations in policy formation for a range of healthcare issues [11, 13], yet policies founded on adult message framing may have unintended consequences for adolescents. It is well-known that policy-making processes are rooted in political and public will governed by civil rights, incrementalism, pluralism, and federalism. Developing effective policies is equally challenged by insufficient evidence from research and arguably, appropriate stakeholders that can provide new perspectives to inform decisions. To minimize marginalization of adolescents and exercise equity in the provision of relevant policy interventions and initiatives that may optimize uptake, the purpose of our study was to explore lifestyle treatment recommendations from Canadian Anglophone and Francophone adolescents with obesity to inform policy and program decisions.

## Methods

### Design

This qualitative, cross-language, patient-oriented study was completed between July 2017 and January 2018 and is part of a larger study designed to identify the factors that impact the adoption of lifestyle treatment goals by adolescents with obesity. Our report employs a qualitative description lens as described by Sandelowski (2000) [14]. We also completed a preliminary step consistent with principles of patient-oriented research [15] to engage adolescent patients (as partners) in the design and planning of our study (please see Kebbe et al. (2018) [16] for details). We obtained human research ethics boards and operational approvals for study sites in Edmonton, AB and Ottawa, ON, including the University of Alberta, Alberta Health Services, and the Children’s Hospital of Eastern Ontario. We adhered to the Consolidated criteria for reporting qualitative studies (COREQ) (please see Additional file 1).

### Participants and recruitment

Adolescents (13–17 years of age, body mass index [BMI] ≥85th percentile) were sampled purposefully from one of two multidisciplinary weight management clinics: Anglophones from the Pediatric Centre for Weight and Health (PCWH; Stollery Children’s Hospital, Edmonton, AB) and Francophones from the Centre for Healthy Active Living (CHAL; Children’s Hospital of Eastern Ontario, Ottawa, ON). Both of these clinics are located at children’s hospitals in urban areas and include multidisciplinary teams composed of exercise specialists, dietitians, mental health professionals, nurses, pediatricians, and social workers. After an initial assessment by all members of the team, long-term support is provided for families either in-person (one-on-one or group sessions) or remotely (videoconference), and includes patient-centered, family-based, and multi-component tailored interventions including dietary, physical and sedentary activities, sleep, and behavioral modifications. These clinics are open to diverse communities, which allowed us to gain a better representation of adolescent experiences from Anglophone and Francophone communities in Canada that may vary by language and culture. Adolescents were eligible to participate if they *(i)* had been active for ≥3 months at the corresponding clinic to ensure that they completed initial assessments and gained some experience in, and understanding of, lifestyle change and *(ii)* did not present with known developmental disabilities. Adolescents were informed about the study using recruitment posters displayed in clinic waiting rooms or approached by clinical and research staff members in-person or by telephone. We scheduled interviews at the respective clinics according to participant availability. We offered adolescents a $25 (CDN) Visa gift card as a token of appreciation.

### Data collection

Prior to data collection, the first author (MK) collected opt-in written consent from parents as well as consent or assent from adolescents based on provincial regulations for age of consent. MK then collected adolescents’ demographic (e.g.*,* date of birth, gender) and anthropometric (e.g.*,* height, weight) data from medical records for descriptive purposes and conducted one-on-one, in-person, semi-structured in-depth interviews (30–60 min in length) with adolescents in either English (in Edmonton) or French (in Ottawa); interviews were conducted on-site to ensure adolescents remained in a comfortable and familiar setting. The interview guide (Table 1) was conceptualized (MK) and reviewed by team members with methodological and/or content expertise (AB, SS, TLM, GDCB, Patient Engagement Panel). Interview questions were informed by a previous scoping review of the literature that our team conducted pointing to unique multisector influences on the adoption of healthy lifestyle habits among adolescents with obesity, including the home, school, broader community environment, and policy [10]; each question in the interview guide was accompanied by probing questions to elicit deeper responses on adolescents’ recommendations, including a final round of open-ended discussion for feedback on interview quality and content. We also asked adolescents to select their own pseudonyms to help readers follow individual narratives [17]; if participants were indifferent, we chose names likely to resonate with their identity [18]. MK documented field notes and memos immediately after the interviews, and all interviews were digitally-recorded and uploaded to an online and secure file sharing platform (LabKey) maintained by the Women and Children’s Health Research Institute (UAlberta).

**Table 1:** Interview guide exploring recommendations from adolescents with obesity to adopt healthy lifestyle behaviors

1. Do you ever feel like there are external factors that aren’t in your control that influence your lifestyle choices?	
2. What kinds of changes can we make to help teens have a healthy lifestyle?	
Family setting	
3. Some teens say that their family affects their lifestyle (like in the way you eat, exercise, sleep, and emotionally feel). Would you say the same about yours? *(If yes)* What changes do you think your family could make to help?	
School setting	
4. What about your school? Is there anything that schools can do to help you make healthy lifestyle choices?	
Community setting	
5. In terms of the place where you live, what do you think that your community and city can do to help you make healthy lifestyle choices?	
Clinical setting^a^	
6. You’ve been receiving care for your health and weight at this clinic. What’s it like to come here? Is there anything that you like?	
7. Is there anything that you don’t like about going/coming to this clinic?	
8. How can we change things at this clinic to help you better?	

^a^Clinical setting recommendations by adolescents with obesity were all in relation to decision-making preferences and were beyond the scope of this report

### Data analysis

We performed descriptive analyses (means) to characterize our study population. Qualitative data were transcribed verbatim (*Translation Agency of Alberta*), translated (if applicable – please see Kebbe et al. (2018) [16] for details), and verified for completeness and accuracy for analysis. We used *NVivo 11* (QSR, Melbourne, Australia) to manage the data. Analyses of de-identified transcripts were completed independently by two research team members (MK and AP) using qualitative and quantitative manifest and latent content analysis [19], with input from CR on French data. Development of the coding tree included familiarization with each interview as the unit of analysis, iterative development and refinement of codes, sorting and abstraction of text into data-developed categories, and reflexive team discussions. We used several verification strategies to enhance rigor of our study, including investigator responsiveness, methodological coherence, sampling adequacy, and theoretical thinking [20]. Sampling adequacy was determined by data saturation and recommendations by Kvale and Brinkmann (2009) for 15 ± 10 qualitative interviews to yield information-rich data from participants [21].

## Results

A total of 19 adolescents participated in our study, most of whom were female, Anglophone, Caucasian, lived with severe obesity, and had parents who met criteria for overweight and obesity (Table 2). Adolescents’ recommendations were combined across clinics since the categories identified from the transcripts were largely overlapping; these included: *(i)* establish parental support within limits, *(ii)* improve accessibility and availability of ‘healthy foods’, *(iii)* limit deceptive practices in food marketing, *(iv)* improve accessibility and availability of varied physical activity opportunities, and *(v)* delay school start times. A summary with key messages for practical measures is provided in Table 3.

**Table 2:** Demographic, anthropometric, and sociodemographic characteristics of adolescents and their parents

	Adolescents (*n* = 19)	Parents (*n* = 19)
Age (y)	15.1 ± 1.7	49.5 ± 9.0
Sex (n; %)		
Female	11; 57.9	13; 68.4
Male	8; 42.1	6; 31.6
Ethnicity, (n; %)		
Caucasian	13; 68.4	13; 68.4
Non-Caucasian	6; 31.6	6; 31.6
Education (at least college or university) (n; %)	–	10; 52.6
Household Income (>$50,000/y CDN) (n; %)	–	13; 72.2^a^
Height (cm)	164.7 ± 7.0	164.9 ± 11.0
Weight (kg)	103.8 ± 16.7	83.7 ± 14.4
Weight Status (n; %)		
Normal Weight	–	1; 5.3
Overweight	–	10; 52.6
Obesity	4; 21.2	6; 31.6
Severe Obesity	15; 78.9	2; 10.5
Body Mass Index (BMI; kg/m^2^)	37.9 ± 4.1	30.8 ± 5.2
BMI percentile	99.9 ± 0.001	–
BMI z-score	3.5 ± 0.6	–

Data presented as mean ± standard deviation unless otherwise specified

^a^*n* = 18; one parent chose ‘prefer not to say’

**Table 3:** Key messages for practical measures per category and sub-category of recommendations

Category	Sub-Category	Key Messages for Practical Measures
Establish parental support within limits (*n* = 15)	Minimizing family conflict and involvement	• Preference for non-authoritarian-style parenting and value of independence • Negative impact of family conflict on mental health
	Modelling active support by parents	• Desire for parent role modeling for increased motivation • Desire for support in health-related decisions and changes, including nutrition such as preparing healthy leftovers and pre-packaged meals; physical activity such as providing encouragement in being active; sedentary activity such as permitting use of digital technology while exercising; and sleep such as providing bed time reminders
Improve accessibility and availability of ‘healthy foods’ (*n* = 14)	Economizing healthy foods	• Lowering cost of healthy foods to increase motivation for healthy eating • Removing tax on healthy foods to promote a healthy lifestyle
	Implementing school-wide changes	• Providing healthy food options in cafeterias • Facilitating enrollment in home economics classes and access to recipe books with healthy food ideas • Removing restrictions on food consumption in classrooms
Limit deceptive practices in food marketing (*n* = 6)	Changing the digital food environment	• Impact of digital editing of fast food advertisements to look more appealing • Television advertisement tactics of airing a higher proportion of unhealthy than healthy food commercials, and the need to reverse this trend or ban commercials of unhealthy foods
	Changing the commercial food environment	• Promoting environmental restructuring • Showcasing healthy items as opposed to junk food in supermarket aisles and near cash registers • Removing public vending machines to limit access to sugar-sweetened beverages
Improve accessibility and availability of varied physical activity opportunities (*n* = 12)	Implementing school-wide changes	• Better dissemination of physical activity opportunities to facilitate meeting physical activity requirements • Simplifying access to school facilities for physical activity during non-supervised times • Limiting the degree of monitoring done by authority figures during supervised times • Balancing sports with fitness/running classes by merging Sport Performance and Physical Education classes
	Implementing community-wide changes	• Tailoring physical activity opportunities to different ages, religions, and interests • Facilitating access to physical activity opportunities by revisiting cost and distance issues
Delay school start times (*n* = 4)	Delaying school start times	• Influence of lack of sleep on unhealthy dietary and physical activity choices • Delaying school start times by approximately 1 hour

### Establish parental support within limits (*n* = 15)

Adolescents perceived their parents as key players in helping them make lifestyle changes. Importantly, they did not express support for authoritarian-style parenting, which is well-known to be inferior to authoritative-style parenting on a number of health outcomes. Dominic shared that within a family, “*You can’t always control what is happening. You can try to give your opinion, but it won’t really work. It’s the adults that come first, you’re forced to listen to them.”* Chloe added, *“Like, my Mom’s constantly breathing down my neck and I’m like, give me some space sort of thing.”* These behaviors, which adolescents labelled as counterproductive, may not only have undermined adolescents’ evolving abilities, but sometimes resulted in increased family conflict, thereby influencing adolescents’ mental health. Nixy shared her perspective:

“*I want to run away. I can't handle it with my family anymore. I don't like having to deal with them all the time because they fight a lot and then, like, my Dad gets mad easily and then starts fighting with me and then my Mom always gets pissed off at me for nothing, and I guess, like, I cry a lot because of them, so I guess just it's bad for, like, my health.”*

Many adolescents, including Chloe, described parents shifting the blame on their sons and daughters, and vice versa, which is a scenario that tends to be observed in practice to a similar extent. For example, adolescents commented on lack of active involvement by their parents in making changes and associated impact on personal motivation levels. Respectively, Chole and Mona shared their experiences:

*“I kind of feel like my Mom looks for every single little flaw that kind of comes out and they [parents] don't really exercise a lot with me. So, I feel like I don't have enough motivation and they're saying, oh yeah walking the dog isn't enough and stuff, so I guess that doesn’t really help with kind of getting along. My Mom’s trying to lose weight, but my Dad’s not, so it’s like, it's not an entire family sort of thing household and my brother that's still living at home, he's kind of really slacking off somewhere, not doing much or always out, so that doesn't help either.”*

*“Well, they are already making some changes anyways, so... but my dad is not really involved, because in my case, my parents are separated. So, I don’t talk to my dad, so he can’t realize what is changing in my life. My mom helps me, my step-dad also helps me, he is super cool.”*

To resolve these issues, but still maintain a degree of independence in light of increasing developing autonomy and identity, adolescents recommended parents to practice role modelling and actively support their attempts, within limits, at a healthier lifestyle. Nicholas stated:

*“I am starting to do things on my own, but not in relation to health or politics, I prefer they [parents] come with to advise me, but I am starting to realize that I like social situations where I come alone, I prefer doing things alone, but I am happy that they’re here to support me in things like health care.”*

Other adolescents echoed Nicholas’ preference for parental involvement in health-related decisions, and provided recommendations particularly for nutrition (e.g.*,* preparing healthy leftovers and pre-packaged meals), physical (e.g.*,* providing encouragement) and sedentary (e.g.*,* permitting digital technology while exercising on the treadmill) activities, and sleep habits (e.g.*,* providing bed time reminders). For example, Daniel explained, *“I need an internet connection and a phone because then I can watch YouTube while I’m walking [basement treadmill].”* and recommended his parents facilitate and support this combined activity. These examples showcased adolescents’ desire for achieving optimal balance in parents’ involvement in their lives, and can be of great importance to inform the conceptualization of lifestyle treatment interventions.

### Improve accessibility and availability of ‘healthy foods’ (*n* = 14)

Adolescents commented on both accessibility and availability of healthy foods in their immediate and broader environments. A number of adolescents reported that cost was a determining factor of their eating choices, and most recommended lowering the cost of healthy foods to help them make healthier purchases more often. Michel linked less costly foods with increased motivation levels to eat healthy: *“The cost, if you find something that is healthier and less expensive. These things will motivate you to eat healthy.”*

On how to achieve this change, Nicholas and Dominic described their perceived – yet imprecise – understanding of the provincial taxation systems in Alberta and Ontario, respectively, and recommended, but also questioned, the impact of any changes:

“*And then, I find that it’s good that the government is putting a Carbon tax, but they could remove tax on what is healthy and organic, what’s good for us. So, meats, vegetables and so, they could remove tax on that since it would promote a healthier life that would give better health to people.”*

“*It’s just making a change to our tax and it’s this or having sales or things like that, it will just help us, but it’s still expensive. It’s really expensive to buy fruits and all that. It’s less expensive to buy chips or something else other than fruits.”*

Despite having access to a dietitian as part of multidisciplinary clinical care for pediatric weight management, one adolescent made a distinct recommendation to facilitate access to healthy foods, namely enhancing resources (e.g.*,* recipe books) that emphasize healthy, budgetfriendly foods. Courtney supported her recommendation with an anecdote:

*“More programs telling families like here are some recipe ideas that are healthy, but like really cheap or even a Facebook page or something because it’s really hard planning a meal that’s different every night that’s healthy, but isn’t like extremely expensive to plan. Right now, we’re trying to keep our grocery bill to like a hundred and forty every Sunday, but it has to feed four people and that also has to include buying animal food when we need it because we have six pets. My cousin [...] they actually have a book that they distributed at schools that was like a little recipe book, an idea book and stuff. So that was really useful.”*

Although such resources were perceived to be helpful by Courtney, a few others commented on personal risk-benefit ratios of the cost of healthy foods and preparation time; instead, they recommended having greater availability of healthy foods that they could eat in different settings and on the go to accommodate their busy schedules. The belief of longer preparation time of healthy foods, as noted by Dominic below, tends to be observed among the general population, and may be indicative of established habits, a lack of motivation, or a lack of self-efficacy and skills in culinary preparations.

*“You buy a salad and it’s like $10 and you could prepare it yourself, and it will cost maybe $5. Now, teens, I feel... it’s just easier to go to McDonalds or Subway or similar fast food chains. It’s easier to buy fast food than to prepare your own salad or sandwich or something like that.”*

When asked about nutrition in the school setting, adolescents initially reciprocated with indifference. However, on further discussion, it became clear that nearly all adolescents viewed their school cafeterias (if available) as roadblocks to a healthy diet and recommended healthier alternatives. It is worth noting that the recounted imbalance of healthy foods in favour of unhealthy foods in school cafeterias may explain why most adolescents’ recommendations were to limit unhealthy foods rather than increase availability of healthy foods; the impact of this imbalance on adolescent food choices is a subject of speculation. Consider the examples Nicholas provides below:

*“The cafeteria isn’t really healthy either, they’re ‘fast food’ cafeterias, it’s pizza, it’s small noodles that you freeze, it’s sandwiches, small wraps, it’s not necessarily healthy. You can have healthier options, you can have salad, you can have… it’s pretty much all, you can just have salad that is good. So, there needs to be a big change at school, but school can’t do anything because it’s the Minister [of Education] and then, the minister doesn’t want to change.”*

Unlike most affirmations for change, one adolescent did not perceive cafeteria food, except for fries, as being unhealthy, and shared no recommendations for change in this environment: *“I don’t think it’s unhealthy, so...”* (Abdi). This raises the question of how and why adolescents attribute particular indicators and dimensions to healthy and unhealthy foods. Others, such as Dominic, speculated about the practicability of any changes:

*“My cafeteria has changed so often. When I started in 9*^*th*^ *grade, way before it was all poutines, pizza, fatty things. After, it changed to be healthy, salad, chicken, things like that. After, it changed again and they re-added the things [unhealthy foods] to make money, because everyone was buying that. But now, they put a mix of both. I mean it’s good for health, there’s other things [than unhealthy foods], but it’s hard to change things at school [for the better]. There’s still vending machines, there’s still things like that, but to change things at school [for the better], they try each year and it doesn’t work.”*

A few adolescents shared that classes at school (e.g.*,* home economics) focused on healthy eating would be helpful to build practical knowledge, skills, and culinary techniques. However, enrolling in nutrition education classes at school was not without challenges; some adolescents expressed difficulties due to the popularity of these classes among students and limited available spaces, which is contrasted with vanishing home economics classes in many jurisdictions. Eliza notes:

*“My high school had foods class, and it’s almost like you don’t have a chance to take it. As soon as it opens, you’re placed on a waiting list to enroll in the class [...] maybe they should have had a second class.”*

Outside of home economics, adolescents described how they were not allowed to bring food into their classrooms. Some adolescents like Abdi shared negative views around this issue: “*At school, in my classes… in my English and art classes, we’re not allowed to bring food, even if we’re hungry, they say no and then, I think it’s stupid, because if I’m hungry, I will eat. So…”.*

Adolescents reported that this restriction negatively affected their eating later in the day. Therefore, they recommended that schools do more to accommodate students in this regard, which may be of importance since proper response to hunger and satiety cues can positively manipulate weight regulation.

Overall, adolescents unequivocally indicated being supportive of change for increased accessibility and availability of healthy foods, but this was conflicted scepticism on the implementation of policy changes. The conversation around food regulation revealed that some adolescents were unable to recognize existing tax structures and subsidy options on zerorated supplies nor correctly operationalize healthy and unhealthy foods. Worth noting is that the adolescents who voiced these economic concerns did not comment on access to home economics or other food education interventions, which may be worth revisiting in spite of unavailable evidence as a direct effects model. That is, a stimulus-response relationship between food education classes and knowledge on food and food policies.

### Limit deceptive practices in food marketing (*n* = 6)

Adolescents commented on the extent to which digital and commercial culture saturated their lives and highlighted the need to change the digital and commercial food environment to facilitate dietary changes. As Michel stated: *“We should do something. It’s [access to unhealthy food environment] made too easy.”* Specifically, adolescents reported being aware of tactics used in digital food advertising, such as airing a high proportion of unhealthy to healthy food commercials on television and social media sites (e.g.*,* YouTube). Adolescents’ recommendations included airing more commercials of healthy foods and banning commercials of unhealthy foods. As stated by Amber, *“Always, when they show the commercial, usually every single commercial is about junk food. And then you’ll see the one or two like special like good foods for you. That’s pretty much it. They should fix that.”* Nixy provided support for these recommendations by commenting on the impact of food advertisement on her appetite*:*

*“Commercials for junk food make me hungry then I want food and then I go try to find food and I can’t find food so then I just drink chocolate milk. It’s repetitive. I just want commercials to stop, they’re annoying.”*.

Adolescents described other tactics used in food advertising, including the digital editing of fast food advertisements to make them look more appealing. As Dominic noted: *“They always want to increase their revenue, they want to show how food is. It’s not always like in the picture. They show you a picture, it’s not really that, I mean…”*

Eliza described an exaggerated example of digital editing of food that is consistent with societal expectations surrounding women’s bodies:

*“I watched a video with Photoshop, where they took a picture of a slice of pizza and made a perfect girl. It looks like a girl, but when they undid the picture and all the Photoshop, it was a slice of pizza [...] so, why do you need a model that has 6 feet of legs and the size of a twig or tooth pick when you can make a girl out of pizza. I saw this and I assure you I was mind blown.”*

She continued, *“fruits, and other foods that are like the best for you, there isn’t a lot of packaging. It’s just fruit. Then if it’s processed, it’s like super [colourful], it’s really in your face.”*

Another element of food marketing that adolescents commented on was in relation to advertising manipulation in the commercial environment, which incited adolescents to make recommendations for environmental restructuring. Several adolescents noted promotion of junk foods in numerous aisles or in attractive places like near cash registers in supermarkets, and how they perceived this to be done strategically. As Nicholas stated:

“*Here, I am thinking not only for the health of people, but also about the health of the planet. When you put all this candy near the cash register or when passing by checkout, you’re promoting more processed things that take time to produce, that need more resources I should say, natural resources and so they are less expensive. I would change these promotions to advertise items on sale that are healthy for the environment and us as well.”*

Similarly, a few adolescents remarked the use of vending machines as a manipulative sales platform, particularly for sugar-sweetened beverages, and how these interfered with other planned activities; whether this trigger is equally observable among adolescents without obesity and how it influences their subsequent behaviors is unknown. For example, Daniel suggested to remove vending machines from his local swimming pool:

*“With diet, probably make it so that there’s no pop vending machines at the pool because it’s easy for me to just get like two dollars and twenty-five cents and just go there and buy a pop and not actually go swimming.”*

Of note, the complexity of digital and commercial environments calls for more robust media and food literacy education as these too, may have influenced adolescents’ understanding of nutrition and lifestyle, and subsequently their recommendations. For example, while most adolescents correctly labelled fast foods as unhealthy and fruits and vegetables as healthy, a few adolescents incorrectly placed bagels and other cafeteria food in the healthy category and fruits in the unhealthy category due to their sugar content. Lack of knowledge on the digestive physiology of dietary fiber found in fruits and postprandial glycemic responses to sugar in fruits compared with other sources of sugar was alluded to by an adolescent who compared the sugar content of fruits and vegetables, and stated that “*It [sugar content] depends on the fruit. Vegetables aren’t as bad, they have more nutritional value than fruits.”* (Mona). Dominic expanded on other likely drivers of this misclassification, *“A lot of people have complained that fruits aren’t natural, they add chemicals to them, which means less fruits and vegetables were being purchased, which means less uptake in supermarkets.”*

Collectively, adolescents’ discussions of food marketing revealed vulnerabilities to marketing which should be taken into account when considering regulations for digital and commercial environmental restructuring. Of note, while adolescents argued that marketing was often misleading, they did not comment on their logic behind supporting food regulation as a solution.

### Improve accessibility and availability of varied physical activity opportunities (*n* = 12)

Although some adolescents reported being aware of available physical activity opportunities at school and in their communities, others lacked information and recommended that opportunities be better disseminated to reach more adolescents. Those who were aware of opportunities commented on limited choices and variety of physical activity and recommended having physical activity opportunities tailored to different ages, religions, and interests. In support of this recommendation, adolescents discussed how the currently available physical activity programs were mostly designed for younger boys and girls:

“*And so, I would add more physical activity programs that are not only accessible for the community, and kids, but also teens. Because right now, it’s programs that are for kids.”* (Nicholas)*.*

Another adolescent recounted a time when her parents did not allow her to join organized physical activities with religious components:

*“Um, it would be cool if there were some more like uh teen programs that weren’t religion-based.”* (Courtney)*.*

These issues are reminiscent of a dominant parenting style and likely hindered finding proper physical activities to partake in to fulfill physical activity requirements for one of her school classes:

*“It had to be volunteering for ten hours and it had to be supporting physical activity, and so the main areas where they needed volunteers were like for when marathons were being run, but that’s only two hours at the most, so then it’s like great – now I need five more marathons somehow. So that made it really challenging. I checked with my high school and then checked with my sister’s junior high, and mostly just looking in the newspaper and Saint Albert’s has a website where you can see where they need help volunteer-wise, and they just didn’t really have anything. Yeah, so that was like a challenge with that so I just dropped the course because it was too stressful.”*

Adolescents, including Courtney, also desired more flexibility in exercising at school, such as allowing flexibility in access to facilities during non-supervised times and limiting the degree of monitoring being done by authority figures during supervised times. Of note, some adolescents described a benefit to coaching by a fitness instructor, however preferred to not be monitored by their parents in after-school activities, which highlighted the narrow window of adolescents’ desire for receiving parental support and gaining individual autonomy. Courtney’s recommendation was as follows:

*“For activity, it’d be nice if they had, I don’t know, just like a class where kids come in and kind of go in and do whatever they wanted activity-wise because they [school] have like a really big gym, so if there’s like a set time in the schedule for that to happen, but it wasn’t like gym class where they were telling you what to do kind of, you know? Like that would be really helpful.”*

For classes that did include supervision, adolescents described a preference for sports (e.g.*,* volleyball) over physical education (i.e.*,* focus on fitness and running) classes. Dominic and several other adolescents recommended merging the two classes:

*“I went for the other one [Sport Performance], but the only thing to change is to just put the two together. Do both, workout [Physical Education] and gym [Sport Performance] together, you don’t always have to play a sport, you can do both. This is what I would change.”*

Adolescents also recommended physical activity opportunities be made more accessible, citing issues related to cost and distance. Adolescents shared strong feelings about improving affordability of physical activity programs, recreation centres, and exercise equipment. As Abdi mentioned, “*If someone is trying to lose weight and you sell them something [sport equipment, gym membership] for $50, it’s not right.”* Further, adolescents, especially those who lived in remote areas, noted that most activities were often not local to where they lived and advocated for closer proximity of facilities:

*“Everything else is in the city. Tennis, squash, I’m thinking of what else… swimming. It’s all in the city.”* (Nicholas)*.* They reported that distance would deter them from being physically active, even if they had a way to travel there, which is reminiscent of the earlier observed scenario on consumption of healthy foods being counteracted by efforts and time required for preparation: “*It’s a drive [gym], I could bike there if I wanted to, but it’s a no.”* (Chloe)*.*

In sum, adolescents articulated a number of recommendations to improve physical activity provision, uptake, and sustainability, which was indicative of a disconnect between what is accessible and available for adolescents and their own agendas. That is, local, low-cost, enjoyable, and tailored opportunities to be active.

### Delay school start times (*n* = 4)

Adolescents commented on the complex interrelationship between lack of sleep and their other lifestyle habits. In particular, adolescents described impact on their dietary (e.g.*,* skipping breakfast) and physical activity (e.g.*,* decreased participation) choices due to feeling fatigued as a result of waking up early for school. When discussing sleep, adolescents described favouring later school start times to better match their sleep schedules. As such, adolescents made specific recommendations to delay school start times by approximately 1 hour. Courtney exclaimed:

*“I'd probably change so that I didn't start until like nine in the morning kind of thing, like kind of have a spare first thing because then I wouldn't have to get up until seven thirty whereas right now I'm getting up at like six thirty and this is only my fourth day of school, but I'll need to start getting up sooner so I can like do everything.”*

As Nixy simply reiterated, *“I just, like, I want more sleep.”* Given current regulations for fixed schedules in most schools, adolescents’ earlier recommendations for their parents to provide bed time reminders may be relevant in this regard.

Remarkably, in all categories, a few adolescents reported individual responsibility for making choices in their current environments, yet not in influencing change. As Dipti and Michel described, respectively, in the context of the home, social, and built environments, *“So I guess it’s really the choice of the customer.”* and *“Well, it depends, it’s not really up to me to decide, but if I wanted to see change, it’s to have more healthier options.”* It is possible that adolescents developed ambivalence about a system that they believed had failed them, resulting in perceived selfresponsibility for change. Nevertheless, these excerpts indicated that while these adolescents provided concrete recommendations for an environment conducive of a healthy lifestyle, they were equally unconvinced of ensuing changes in the context of both personal and policy actions.

## Discussion

To our knowledge, there have been no studies from the perspective of adolescents with obesity to explicitly examine lifestyle treatment recommendations construed as a public health issue. Our study explored lifestyle treatment recommendations made by Anglophone and Francophone Canadian adolescents with obesity to inform policy and program decisions. Our findings suggest that parenting dynamics, collective framing of our food environment, accessibility and availability to physical activity opportunities, and sleep habits were perceived as key targets for a healthy lifestyle. Recommendations covered a variety of topics for multiple settings and stakeholders, which highlights the need for system-oriented multi-level interventions to potentially impact adolescent health behaviors.

The experiences of adolescents in our study add to our understanding of the inherent challenges that they encounter within an environment most familiar to them – the home. Adolescents’ recommendations emphasized a supportive home environment with less parental conflict. Previous studies and reviews have shown links between youth weight and household organization, including sleep, screen time, and family meal routines [22, 23], highlighting parents as drivers of adolescents’ lifestyle behaviors and their unique role in the establishment of a health-promoting environment. Specifically, adolescents wanted their parents to act as role models by implementing healthy lifestyle changes in nutrition and physical and sedentary activities. The advantages of role modelling are supported by well-known behavior change theories (e.g.*,* the Social Cognitive Theory) [24] and are supported by recent work by Berge et al. (2013) [25]. Authors characterized parental influence on adolescents’ food behaviors; they found that adolescent girls were less likely to diet, binge eat, and attempt to control their weight in unhealthy ways if their parents engaged in healthy eating themselves. Interestingly, while adolescents with obesity recommended parental efforts to act as positive role models for both healthy nutrition and physical activity changes in our study, it has been reported that they may be more receptive of encouraging comments for adopting healthy physical activity than nutrition habits [16]. It is possible that adolescents may perceive an individual responsibility to change nutrition habits, compared with physical activity changes that may benefit from reminders and encouragements due to a reported lack of motivation [10, 26]. These outcomes are partly consistent with longitudinal research showing positive temporal associations between paternal permissive-style parenting (high responsiveness, low demandingness) on fruit and vegetable intake in female adolescents, yet paradoxical in light of no significant predictions between authoritative-style parenting (high responsiveness, high demandingness) and physical activity in male and female adolescents [27]. Whether these findings are generalizable to adolescents with obesity, overall dietary quality, and specific types of physical activity should be subject of future longitudinal research.

Schools are a natural setting for effective nutrition and physical activity policies since it is where adolescents spend the majority of their day. Although many adolescents may bring food from home, up to 40% of adolescents’ calories are consumed at school, so the quality of foods and beverages available in this setting is crucial in promoting nutrition standards [28]. As noted by adolescents in our study, school cafeterias tended to offer unhealthy food options to students. Indeed, compared with home cooked meals, foods such as those typically offered in school cafeterias tend to be more energy dense and come in larger portions [29], which may affect total energy intake [30] and body weight [31]. It follows that some jurisdictions are considering healthier changes for foods served in schools, including eliminating trans fats, limiting saturated fat, and decreasing total sugar content [32], all of which are consistent with the 2019 Canada Food Guide [33]. Changes of the sort can improve targeted dietary behaviors in children and adolescents [34], yet any significance of dietary quality served on dietary intake, adiposity, and metabolic risk is important and necessitates more investigation in adolescents with obesity specifically.

School environments can also promote or limit healthy physical activity for adolescents. Interventions have shown increased physical activity levels in adolescents in schools once barriers were minimized or removed [35], but as with any intervention, these may suffer in their sustainability. Since public health efforts normally target individuals in isolation of their surrounding social context [36], expanding these across ecological contexts can likely increase sustainability of behavior change over time by helping shift community and organizational norms. A previous qualitative study [37] with regular-weight adolescents on identifying improvements for physical activity provision, uptake, and sustainability revealed highly overlapping recommendations by adolescents with obesity in our study, which highlighted the importance of relevance, choice, and motivation for adolescents as a whole. Consequently, addressing availability, accessibility, flexibility, and dynamics are paramount to improve adolescent activity levels. As for all policy and program decisions, adolescents should be involved in the design and implementation of physical activity interventions to account for their interests and preferences. Previous research has shown positive outcomes of engagement of youth in the development of health care research interventions for both the research (e.g.*,* better understanding of ethical considerations and economic consequences of the interventions) and stakeholders involved (e.g.*,* development of interpersonal and team working skills, empowerment, and empathy, understanding, and satisfaction) [38, 39]. Stakeholder engagement is consistent with patient-oriented research principles [15], and regardless of any changes to intervention effectiveness, remains of intrinsic value.

Adolescents made several recommendations for policymakers regarding food marketing, which is a powerful factor in the health of young people. The potency of our external environment that is in part responsible for the increased food intake observed today has been influenced dramatically by food advertisements [40]. This is evidenced by more than $1 billion annual expenditure on food and beverage marketing that targets adolescents specifically [41] with very little spent on nutrient-dense products [42]. Adolescents in our study commented on the imbalance between unhealthy and healthy food marketing and advertising and shared their concerns on this issue. These were comparable to qualitative work by Elliot (2017) among regular-weight adolescents who affirmed that advertisers deliberately try to mislead consumers [43]. This study, however, differs from ours in that adolescents expressed concern for younger children, and not themselves, as being a target of these manipulations [43]. Although much of the research is descriptive in nature, studies have suggested a positive link between advertisements for unhealthy food on the Internet and in advergames and children’s desire for and consumption of the foods promoted [44, 45]. In an attempt to counter effects on dietary intake, government health agencies established recommendations for parents to limit screen time, including television viewing, to minimize exposure to advertisements [46]. Since this approach may be disputed by adolescents, others have suggested regulating advertisements for unhealthy foods by drawing inspiration from the 1980 Consumer Protection Act in the province of Québec that imposed legislation to prohibit all commercial advertisements to children [47]. Yet, *adolescents* in Canada remain exposed to junk food advertisements while awaiting amendments and approval of a Health Canada legislation to prohibit marketing of unhealthy food to children in other provinces and adolescents under the age of 17 [48]. A similar, discouraging pattern is observed among food manufacturers, with existing policy norms tending to rely on industry self-regulation to limit marketing of foods high in fat, sugar, and sodium [49]. Failed voluntary standards to reduce marketing pressures on adolescents call for the use of legal measures to promote and advocate for healthy nutrition. A notable example is the introduction of an excise tax on sugarsweetened beverages in the United States to disincentivize consumer purchase and reduce consumption. Certainly, roadblocks and political criticisms of these new policies are likely to be ensued, but the promising findings on decreased consumption [50] and rapid international expansion of this taxation [51] cannot be dismissed, especially if tax proceeds are used for health promotion.

Overall, our findings suggest the need to broaden the framing of obesity-related policy to go beyond nutrition and physical activity and target other modifiable lifestyle areas (e.g.*,* sleep) that dictate chronic disease risk, including obesity. The implications of our findings should be considered alongside our study limitations. Arguably of highest importance, our study relied on reported effectiveness, and future research investigating measured effectiveness is essential to complement identified recommendations as trusted strategies. Next, most participating adolescents were of Caucasian origin, and adolescent experiences and recommendations may be varied sociodemographically. To increase transferability of our findings, we included both Anglophone and Francophone participants from two geographically diverse provinces in Canada, thereby strengthening implications on a national level. Lastly, adolescents may have been limited in their ability to articulate some of their experiences and formulate recommendations due to challenges in delving into macro-level factors. To minimize these possibilities, the interviewer provided adolescents with numerous probes and opportunities to refine or expand on their answers at multiple time points during the interview.

In conclusion, our findings indicated that adolescents’ recommendations may not be adequately reflected in current health-promoting lifestyle initiatives and interventions. Identified stakeholders should consult and involve adolescents in policy and program development, implementation, and evaluation to be more responsive of their needs; such an approach is consistent with recommended government roles and actions for a healthier food system [52]. Notably, through a larger study that informed this work, we developed a theory-driven patientcentered clinical tool *with* and *for* adolescents with obesity in order to empower them to be engaged in their own care and make healthy lifestyle changes [53]. Acknowledging the recommendations made by adolescents in our study can improve the uptake, sustainability, and overall success of planned projects.

## Supplementary information

**Additional file 1:** **Table S1.** Consolidated criteria for reporting qualitative studies (COREQ). Reporting checklist for qualitative research.

## Data Availability

All data generated or analysed during this study are included in this published article.
