# Supplementary material for: End-user perspectives to inform policy and program decisions: a qualitative and quantitative content analysis of lifestyle treatment recommendations by adolescents with obesity
Source: BMC Pediatr. 2019 Nov 8;19:418. doi: 10.1186/s12887-019-1749-3 (PMC6839254; doi:10.1186/s12887-019-1749-3)
Supplement: Supplementary file 1 — Additional file 1: Table S1. Consolidated criteria for reporting qualitative studies (COREQ). Reporting checklist for qualitative research. [file 12887_2019_1749_MOESM1_ESM.docx]

**Additional file 1.** Consolidated criteria for reporting qualitative studies (COREQ)

| **No.** | **Item** | **Question** | **Description** |
| --- | --- | --- | --- |
| **Domain 1: Research Team and Reflexivity**  *Personal Characteristics* | | | |
| 1. | Interviewer | Which author(s) conducted the interviews? | First author (MK) conducted interviews in Edmonton and Ottawa. |
| 2. | Credentials | What were the researchers’ credentials? | MK: BSc  AP: PhD  AB: PhD, C. Psych  TLFM: PhD  SDS: RN, PhD  CR: PhD, RD  MPD: PhD  GB: PhD, RD |
| 3. | Occupation | What was their occupation at the time of the study? | MK: Graduate Student  AP: Postdoctoral Fellow  AB: Clinical Psychologist  TLFM: Associate Professor  SDS: Professor  CR: Assistant Professor  MPD: Assistant Professor  GB: Professor |
| 4. | Gender | Was the researcher male or female? | MK: Female  AP: Male  AB: Female  TLFM: Female  SDS: Female  CR: Female  MPD: Female  GB: Male |
| 5. | Experience and training | What experience or training did the researcher have? | MK: Formal training in qualitative methods; completed graduate-level coursework in qualitative research  AP: Formal training in qualitative research; completed graduate-level coursework in qualitative research and thesis studies on qualitative inquiry  AB: Clinical researcher with some experience in qualitative inquiry  TLFM: Conducts original research in qualitative inquiry  SDS: Conducts original research in qualitative inquiry  CR: Basic knowledge of qualitative research  MPD: Basic knowledge of qualitative research  GB: Leads several qualitative research studies. |
| *Relationship with Participants* | | | |
| 6. | Relationship established | Was a relationship established prior to study commencement? | Participants had familiarity with the sites where interviews were collected; however, there were no pre-existing relationships between participants and the interviewer. |
| 7. | Participant knowledge of the interviewer | What did the participants know about the researcher? | Participants were not given information about the interviewer beyond a brief introductory statement at the beginning of interviews that described the interviewer’s role in the study and at the clinic sites. |
| 8. | Interviewer characteristics | What characteristics were reported about the interviewer? | None. |
| **Domain 2: Study Design**  *Theoretical Framework* | | | |
| 9. | Methodological orientation | What methodological orientation was stated to underpin the study? | Qualitative description. |
| *Participant Selection* | | | |
| 10. | Sampling | How were participants selected? | Purposive sampling. |
| 11. | Method of approach | How were participants approached? | By telephone or in-person. |
| 12. | Sample size | How many participants were in the study? | 19. |
| 13. | Non-participation | How many people refused to participate or dropped out? Reasons? | 35 participants passively rejected our invitation to participate. Common reasons for non-participation included timing and lack of interest in the study. No participants dropped out of the study. |
| *Setting* | | | |
| 14. | Setting of data collection | Where was the data collected? | Clinic settings. |
| 15. | Presence of non-participants | Was anyone else present besides the participants and researchers? | No. |
| 16. | Description of sample | What are the important characteristics of the sample? | Anglophone and francophone adolescents (13-17 years old) with obesity (body mass index ≥85^th^ percentile) who had been receiving care at the clinics for a minimum of 3 months. |
| *Data Collection* | | | |
| 17. | Interview guide | Were questions, prompts, guides provided by the authors? Was it pilot tested? | Yes. The guide was improved/refined throughout the data collection process. |
| 18. | Repeat interviews | Were repeat interviews carried out? | No. |
| 19. | Audio/visual recording | Did the researcher use audio or visual recording to collect the data? | Interviews were audio-recorded. |
| 20. | Field notes | Were field notes made during and/or after the interviews? | Yes. Field notes were made immediately following interviews. |
| 21. | Duration | What was the duration of the interviews? | Approximately 30 – 60 minutes. |
| 22. | Data saturation | Was data saturation discussed? | Yes. |
| 23. | Transcripts returned | Were transcripts returned to participants for comment or correction? | No. |
| **Domain 3: Analysis and Findings**  *Data Analysis* | | | |
| 24. | Number of data coders | How many coders coded the data? | Two researchers (MK and AP) coded the data independently. Codes and categories were discussed and refined within the research team. |
| 25. | Description of the coding tree | Did authors provide a description of the coding tree? | Yes. |
| 26. | Derivation of themes | Were themes identified in advance or derived from the data? | Derived from the data. |
| 27. | Software | What software, if applicable, was used to manage the data? | *NVivo* 11. |
| 28. | Participant checking | Did participants provide feedback on the findings? | Passively as part of a follow-up quantitative study. |
| *Reporting* | | | |
| 29. | Quotations presented | Were participant quotations presented to illustrate the themes/findings? Was each quotation identified? | Yes, quotations were included in a separate table. Quotations were identified by category. |
| 30. | Data and findings consistent | Was there consistency between the data presented and the findings? | Yes. |
| 31. | Clarity of major themes | Were major themes clearly presented in the findings? | Yes. |
| 32. | Clarity of minor themes | Is there a description of diverse cases or discussion of minor themes? | Both major and minor themes were discussed. |
